# Effects of an increase in population of sika deer on beetle communities in deciduous forests

**DOI:** 10.3897/zookeys.625.9116

**Published:** 2016-10-19

**Authors:** Taichi Iida, Masashi Soga, Shinsuke Koike

**Affiliations:** 1 Graduate School of Agriculture, Tokyo University of Agriculture and Technology, 3-5-8, Saiwai, Fuchu 183-8509, Japan; 2 School of Engineering, The University of Tokyo, 7-3-1, Hongo, Bunkyo, Tokyo, 113-8656, Japan

**Keywords:** Ecosystem functions, ecosystem management, forest ecosystems, herbivores overgrazing, species traits

## Abstract

The overabundance of large herbivores is now recognized as a serious ecological problem. However, the resulting ecological consequences remain poorly understood. The ecological effects of an increase in sika deer, *Cervus nippon* Temminck (Cervidae), on three insect groups of beetles was investigated: ground beetles (Carabidae), carrion beetles (Silphidae), and dung beetles (Scarabaeidae and Geotrupidae) on Nakanoshima Island, Hokkaido, northern Japan. We collected beetles on Nakanoshima Island (experimental site) and lakeshore areas (control site) and compared the species richness, abundance, diversity index, and community composition of beetles between the sites. Results showed that although both species diversity and abundance of carabid beetles were significantly higher at the lakeshore site, those of dung and carrion beetles were higher at the island site. It was additionally observed that abundance of larger carabid beetles was higher at the lakeshore site, whereas that of small-sized carabid beetles did not differ between the lakeshore and island sites. For dung beetles, abundance of smaller species was higher at the island site, whereas that of large species did not differ between the lakeshore and island sites. Abundance of two body sizes (small and large) of carrion beetles were both higher at the island site. Overall, the findings of this study demonstrated that an increase in deer population altered the insect assemblages at an island scale, suggesting further changes in ecosystem functions and services in this region.

## Introduction

The overabundance of large herbivores is now recognized as one of the serious ecological issues worldwide, especially in the northern hemisphere (Gill 1992, Côté et al. 2004, Takatsuki 2009). Indeed, there is mounting evidence demonstrating the serious consequences of large herbivore overabundance on forest ecosystems (Takatsuki 2009, Foster et al. 2014). As large herbivores selectively browse palatable species, browsing has negative effects on understory structure and species composition (Rooney 2009, Martin et al. 2011, Tanentzap et al. 2011). Large herbivores additionally alter forest structure (e.g. Kanda et al. 2005, White 2012) by limiting tree regeneration (Akashi and Nakashizuka 1999, Horsley et al. 2003), thereby inducing a cascading effect on animal species (Kanda et al. 2005, Bressette et al. 2012). Indeed, it has been reported that smaller herbivorous invertebrates are negatively influenced by large herbivore overabundance because of interspecies competition for food resources (Gómez and González-Megías 2002, Wheatall et al. 2013). In addition, browsing results in the degradation of habitat for invertebrates and birds (Stewart 2001, Allombert et al. 2005, Chollet and Martin 2013).

To evaluate the ecological impacts of large herbivore overabundance on ecosystems, previous studies have commonly used manipulations with inclusion/exclusion treatments of large herbivores (Goméz and González Megías 2002, Mysterud et al. 2010, Holt et al. 2011, Bush et al. 2012). However, these previous studies have two major limitations. First, as past studies investigated the effects of large herbivore overabundance up to 10 years (e.g. Suominen et al. 2003, Beguin et al. 2011), the effects on ecosystems were unlikely to be completely detected. Indeed, it is established that the effects of browsing by large herbivores on vegetation structure can persist for an extended period (Tanentzap et al. 2011, Nuttle et al. 2014). Second, to understand the responses of ecosystems to perturbation, it is necessary to conduct a large-scale experiment (Carpenter 1998), whereas previous studies have employed relatively small-scale experiments (e.g. Dennis et al. 1998, Gómez and González-Megías 2002; Iida et al. 2016). Nevertheless, field studies that employed a relatively large-scale experiment remain scarce, with most having been conducted in Canadian coniferous forest (e.g. Allombert et al. 2005, Martin et al. 2010).

**Figure 1.**
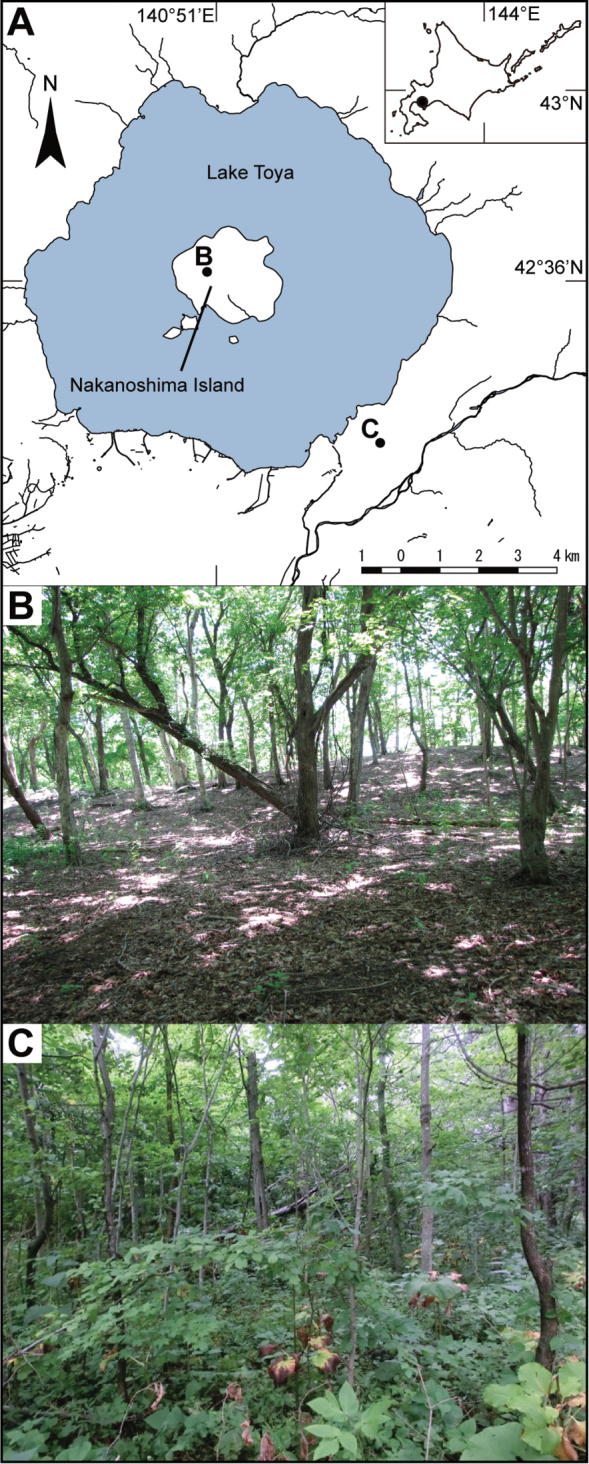
The location and forest floor of the study area. **A** The location of Lake Toya **B** Forest floor of Nakanoshima Island **C** Forest floor of the lakeshore of Lake Toya. The map of Lake Toya was modified from the Geospatial Information Authority of Japan 2015.

Lake Toya located in western Hokkaido, northern Japan, provides an ideal study site to investigate the long-term impacts of deer overabundance on ecosystems (Fig. 1). On Nakanoshima Island (476.7 ha, hereafter, island), which is one of the islands situated within Lake Toya, three sika deer individuals [*Cervus nippon* Temminck (Cervidae)] were introduced approximately 50 years ago and deer density at the island has now reached over 50 deer/km2 (Ikeda et al. 2013). Takahashi and Kaji (2001) reported a significant impoverishment of understory vegetation at the island because of the rapid growth in the deer population (Fig. 1B), suggesting a further consequence for other animal species. In the areas around Lake Toya (hereafter, lakeshore), and at the time of the current study, deer density was approximately 1.1 × 10−2 deer/km2 (Akaba et al. 2014), which is considerably lower than the density at the island site (Fig. 1C). As the island is geographically isolated from lakeshore areas, the island ecosystem could be considered as an experimental site for examining the effects of an increase in deer population on natural ecosystems.

Here, we evaluated the effects of an increase in sika deer population on four taxonomic groups of beetles: carabid (Carabidae), carrion (Silphidae) and dung beetles (Scarabaeidae and Geotrupidae). These beetle groups were selected for several reasons. First, as these beetles inhabit the forest floor and are known to be sensitive to microclimatic changes (Rainio and Niemelä 2003, Arellano et al. 2005), they are likely to sharply respond to the increase in the large herbivores (Rooney 2009, Yamada and Takatsuki 2015). Second, many beetles display species-specific ecological traits, which facilitates the investigation of the association between species life-history traits and their responses to large herbivore overabundance (Sumways 2007, Bachand et al. 2014). Third, the clarification of the responses of beetles to environmental changes is crucial for understanding the overall changes in forest ecosystems, as beetles constitute a large proportion of biomass in forest ecosystems and play important roles that maintain ecosystem functions, such as nutrient cycling and pollination (Kevan and Baker 1983, Speight et al. 2008, Barton et al. 2013).

## Materials and methods

### Study area

Our study area is located in Western Hokkaido, northern Japan. The deer density at the island site (42°36'N, 140°51'E (DDM)) is dramatically higher than that at the lakeshore site because of artificial introduction. The mean annual temperature of this area is 7.3 °C and the mean monthly temperature ranges from −5.1 °C to 20.2 °C. The mean annual precipitation is 984.8 mm and the mean annual snow depth is 30 cm. The study area is situated in a deciduous forest dominated by *Quercus crispula* Blume (Fagaceae); *Kalopanax septemlobus* (Thunb.) Koidz. (Araliaceae); *Magnolia obovata* Thunb. (Magnoliaceae); *Acer pictum* Thunb. (Sapindaceae); *Maackia amurensis* Rupr. et Maxim. (Fabaceae); *Tilia japonica* (Miq.) Simonk. (Tiliaceae) and *Ostrya japonica* Sarg. (Betulaceae) (Kaji et al. 1991). Because of overgrazing at the island site, the structure of understory vegetation and the forest floor differ between the island and lakeshore sites (Fig. 1), with the island site dominated by *Senecio cannabifolius* Less. (Asteraceae); *Sagina japonica* (Sw.) Ohwi (Caryophyllaceae); *Pachysandra terminalis* Siebold et Zucc. (Buxaceae) and *Chloranthus serratus* (Thunb.) Roem. et Schult. (Chloranthaceae), which sika deer find unpalatable (Takahashi and Kaji 2001, Miyaki and Kaji 2009). The forest floor of the lakeshore site is dominated by *Dryopteris crassirhizoma* Nakai (Dryopteridaceae); *Cardamine leucantha* (Tausch) O.E. Schulz (Brassicaceae); *Cephalotaxus harringtonia* (Knight ex Forbes) K.Koch *var. nana* (Nakai) Rehder (Cephalotaxaceae) and *Sasa senanensis* (Franch. et Sav.) Rehder (Poaceae) (observed by authors). Although other large animals including *Vulpes vulpes* Linnaeus (Canidae); *Nyctereutes procyonoides* Gray (Canidae) and *Procyon lotor* (Linnaeus) (Canidae) occur at the lakeshore site, their abundance is negligible compared to sika deer (Akaba et al. 2014). Besides the sika deer, the island site contains no medium- or large-sized mammals (Akaba et al. 2014). We established 30 sampling plots which are 100 m apart from each other (along a 3 km sampling transect) at each of the island and lakeshore sites (42°34'N, 141°54'E (DDM)).

### Beetles sampling

At both sites, we sampled carabid, carrion and dung beetles using pitfall traps baited with cattle dung and fermented milk. Fermented milk is one of the major bates in collecting ground-dwelling beetles in Japan (Suttiprapan and Nakamura 2007). Pitfall traps were constructed using plastic containers (22.5 cm diameter and 26.6 cm deep) and plastic cups (8.3 cm diameter and 11.5 cm deep). Each container of fermented milk was buried to the rim in the ground. Plastic cups containing cattle dung were then hung inside the container using wires. We set plastic roofs on the traps to prevent interference from rain and fallen leaves. These traps were set at a density of one trap per plot. Surveys were conducted during early September 2012 and 2013.

### Species classification

For each beetles group, the insect species collected in the field were divided into different size groups according to body length (see Fujita et al. 2008, Koike et al. 2014). Information on body size was collected from Ueno et al. (1985). Carabid and carrion beetles were divided into groups for small, medium or large species (small, <10 mm; medium, ≥10 and <20 mm and large, ≥20 mm), and dung beetles were divided into groups for small or large species (small, <10 mm; large, ≥10 mm). However, because carrion beetle species which are divided into small size group were not sampled, carrion beetles were divided into the medium or large size groups.

### Data analyses

All analyses were performed using R ver. 3.2.1 (R core team 2015). In order to test the differences in abundance and species richness of each taxonomic group or body length between the island and lakeshore sites, we used generalized linear models (GLMs), using the ‘glm’ function. For GLMs, the abundance and species richness of each taxonomic or body size group were used by a response variable with a Poisson distribution and a log link function. Sampling sites were used as categorical explanatory variables (we used the lakeshore site as a reference). To test the difference in species diversity between two sites, Shannon-Wiener diversity index of each taxonomic group which were calculated by using package ‘vegan’ (Oksanen et al. 2015) were also used as a response variable with a Gaussian (normal) distribution and an identity function. Species diversity indices of each functional group were not calculated and were excluded from the analysis because of the small sample size. Because we could not identify *Synuchus* spp. to the species level, these species were excluded from the analysis of species richness and diversity. We did not find large carabid species in the island sites, which hinders the fitting of our data using GLMs. Hence, we excluded large carabid species in this analysis. Because of small sampling sizes, we used ‘zeroinfl’ function in the package ‘pscal’ for abundance and species richness of small carabid species, and medium and large carrion beetles. We calculated estimated abundance, species richness and Shannon-Wiener indices using the estimates of parameters in GLMs.

To investigate the difference in species composition between the island and lakeshore sites, we performed a non-metric multidimensional scaling ordination (NMDS) using the metaMDS function with the Bray–Curtis measure within the package ‘labdsv’ (Roberts 2015) as well as cluster analysis. The validity of clustering was evaluated using the Calinski–Harabasz criterion. The indicator species value (IndVal) was calculated to identify species, which are indicators of assemblages of each site as illustrated by NMDS and cluster analysis.

## Results

3,876 individuals in total were collected, comprising 824 carabid beetles (18 species), 148 carrion beetles (four species) and 2,902 dung beetles (five species) (see Suppl. material 1).

**Figure 2.**
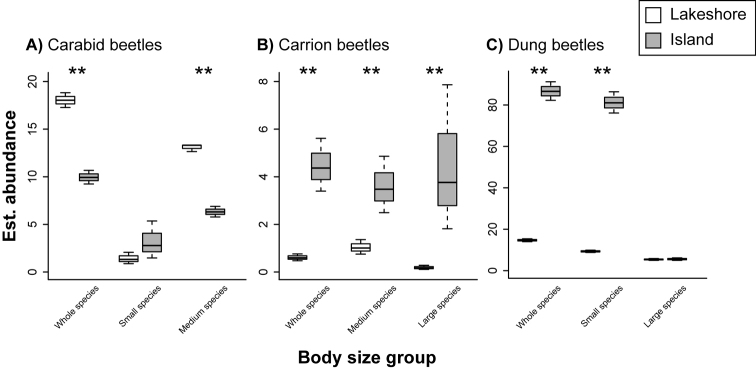
Abundance of each insect taxonomic and functional group per plot at the island and lakeshore sites estimated by using GLMs. **A** Carabid **B** Carrion **C** Dung beetles. Carabid and carrion beetles were classified into small, medium and large species and dung beetles were classified into small and large species (see the main text). Asterisks indicate a significant difference (*p* < 0.05).

**Figure 3.**
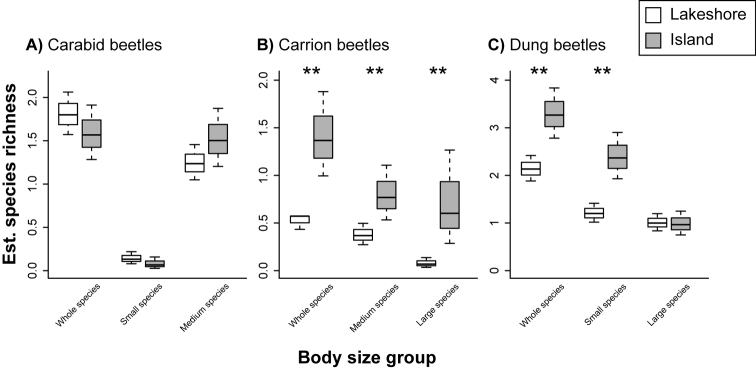
Species richness of each insect taxonomic and functional group per plot at the island and lakeshore sites estimated by using GLMs. **A** Carabid **B** Carrion and **C** Dung beetles. Carabid and carrion beetles were classified into small, medium and large species and dung beetles were classified into small and large species (see the main text). Asterisks indicate a significant difference (*p* < 0.05).

**Figure 4.**
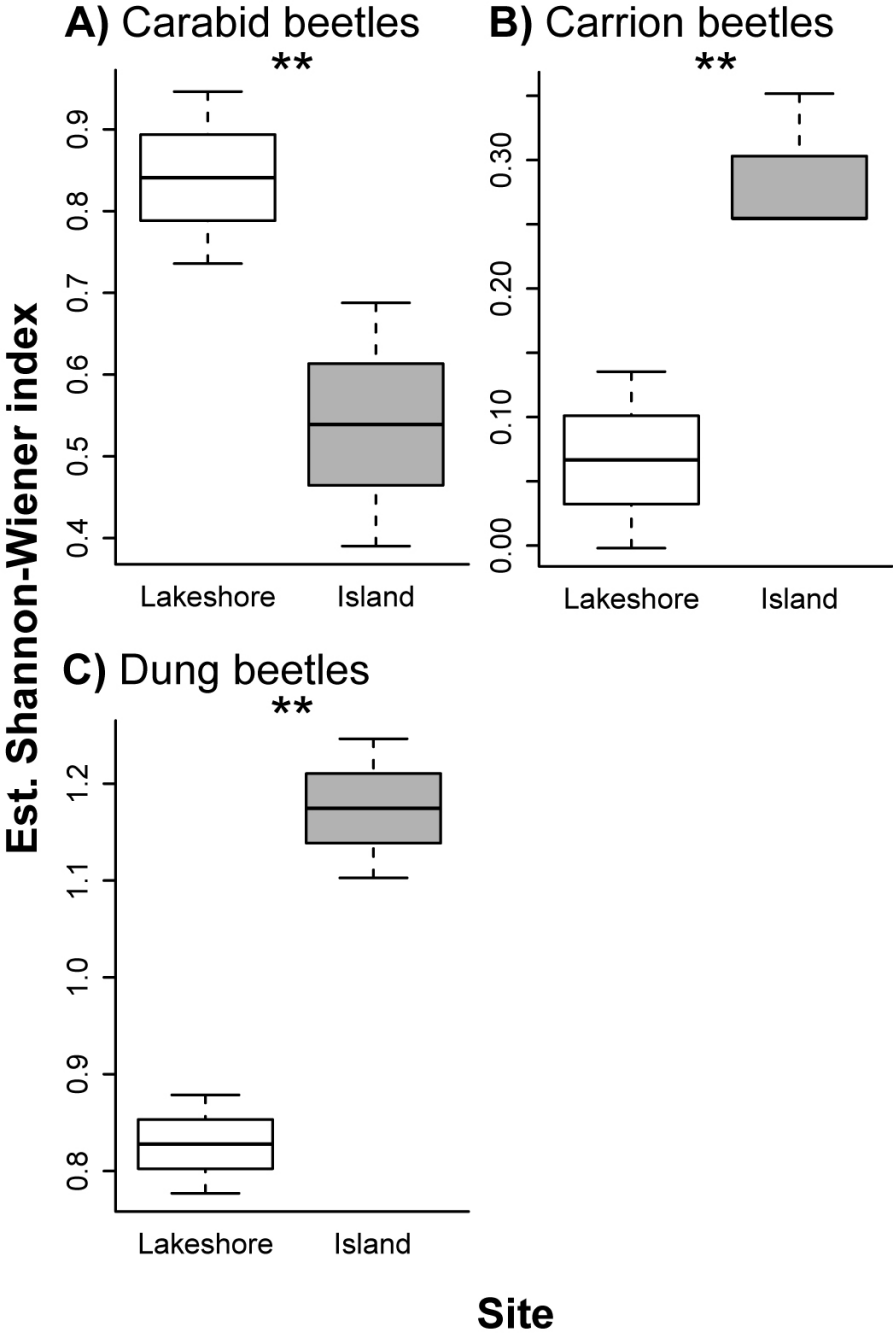
Shannon–Wiener index of each insect taxonomic group per plot at the island and lakeshore sites estimated by using GLMs. **A** Carabid **B** Carrion and **C** Dung beetles. Asterisks indicate a significant difference (*p* < 0.05).

Comparisons between species richness, abundance, and diversity index of insect species between the island and lakeshore sites were summarized in Figs 2–4 and Tables 1–3 (see also Supplementary Tables 2–4 for the differences between estimated and observed values). For carabid beetles, the abundance of individuals at the lakeshore site was higher than that at the island site (*p* < 0.001; Fig. 2A; Table 1). Although the abundance of medium species were higher at the lakeshore site than at the island site (*p* < 0.001), that of small species did not significantly differ between the two sites (*p* = 0.24; Fig. 2A; Table 1). The species richness of all carabid species (*p* = 0.49), small (*p* = 0.42) and medium species (*p* = 0.38) did not significantly differ between the island and lakeshore sites (Fig. 3A; Table 2). The species diversity index was higher at the lakeshore site than at the island site (*p* = 0.05; Fig. 4A; Table 3).

**Table 1.** Estimates of parameter differences between sites (reference = lakeshore site, i.e., coefficients of lakeshore site are zero), standard errors (SEs), z values, and p values in generalized linear models of each of taxonomic group and body size group abundance. Bold letters represent p < 0.05.

	Parameters	Estimates	SEs	z values	p values
Carabid beetles					
Whole species	(Intercept)	2.892	0.043	67.271	< 0.001
	Island	-0.596	0.072	-8.266	< 0.001
Small species	(Intercept)	0.170	0.484	0.351	0.73
	Island	0.799	0.682	1.172	0.24
Medium species	(Intercept)	2.526	0.052	48.911	< 0.001
	Island	-0.707	0.090	-7.864	< 0.001
Carrion beetles					
Whole species	(Intercept)	-0.511	0.236	-2.168	0.03
	Island	1.985	0.251	7.897	< 0.001
Medium species	(Intercept)	-0.051	0.316	-0.160	0.88
	Island	1.280	0.340	3.767	< 0.001
Large species	(Intercept)	-2.226	0.736	-3.023	0.003
	Island	3.536	0.745	4.758	< 0.001
Dung beetles					
Whole species	(Intercept)	2.686	0.048	56.330	< 0.001
	Island	1.776	0.052	34.450	< 0.001
Small species	(Intercept)	2.230	0.060	37.250	< 0.001
	Island	2.165	0.063	34.260	< 0.001
Large species	(Intercept)	1.680	0.079	21.319	< 0.001
	Island	0.031	0.111	0.276	0.78

**Table 2.** Estimates of parameter differences between sites (reference = lakeshore site, i.e., coefficients of lakeshore site are zero), standard errors (SEs), z values, and p values in generalized linear models of each of taxonomic group and body size group species richness. Bold letters represent p < 0.05.

	Parameters	Estimates	SEs	z values	p values
Carabid beetles					
Whole species	(Intercept)	0.588	0.136	4.319	< 0.001
	Island	-0.139	0.200	-0.696	0.49
Small species	(Intercept)	-2.015	0.500	-4.028	< 0.001
	Island	-0.693	0.866	-0.800	0.42
Medium species	(Intercept)	0.210	0.164	1.276	0.20
	Island	0.196	0.222	0.882	0.38
Carrion beetles					
Whole species	(Intercept)	-0.836	0.277	-3.015	0.003
	Island	1.149	0.318	3.609	< 0.001
Medium species	(Intercept)	-1.003	0.302	-3.327	< 0.001
	Island	0.738	0.367	2.012	0.04
Large species	(Intercept)	-2.708	0.707	-3.830	< 0.001
	Island	2.197	0.745	2.948	0.003
Dung beetles					
Whole species	(Intercept)	0.758	0.125	6.061	< 0.001
	Island	0.426	0.161	2.651	0.008
Small species	(Intercept)	0.154	0.169	0.912	0.36
	Island	0.693	0.207	3.348	< 0.001
Large species	(Intercept)	-0.034	0.186	-0.183	0.86
	Island	-0.035	0.265	-0.132	0.90

**Table 3.** Estimates of parameter differences between sites (reference = lakeshore site, i.e., coefficients of lakeshore site are zero), standard errors (SEs), t values, and p values in generalized linear models of each of taxonomic group and body size group Shannon-Wiener diversity index. Bold letters represent p < 0.05.

	Parameters	Estimates	SEs	t values	p values
Carabid beetles	(Intercept)	0.841	0.105	7.995	< 0.001
	Island	-0.302	0.149	-2.030	0.05
Carrion beetles	(Intercept)	0.067	0.069	0.971	0.34
	Island	0.285	0.097	2.935	0.005
Dung beetles	(Intercept)	0.828	0.051	16.300	< 0.001
	Island	0.347	0.072	4.828	< 0.001

**Table 4.** The indicator species values of each species. Bold letters represent *p* < 0.05.

	Taxa	Community	IndVals	*p* values
***Caccobius jessoensis* Harold**	**Scarabaeidae**	**island**	**0.988**	**0.001**
***Onthophagus ater* Waterhouse**	**Scarabaeidae**	**island**	**0.822**	**0.001**
***Eusilpha japonica* (Motschulsky)**	**Silphidae**	**island**	**0.604**	**0.001**
***Silpha perforata* Gebler**	**Silphidae**	**island**	**0.405**	**0.01**
***Pterostichus leptis* Bates**	**Carabidae**	**island**	**0.375**	**0.001**
***Liatongus phanaeoides* (Westwood)**		**island**	**0.345**	**0.001**
*Nicrophorus quadripunctatus* Kraatz	Silphidae	island	0.177	0.23
***Copris ochus* Motschulsky**	**Scarabaeidae**	**island**	**0.172**	**0.02**
*Chlaenius pallipes* Gebler		island	0.115	0.39
*Pterostichus planicollis* (Motschulsky)	Carabidae	island	0.103	0.11
*Pterostichus yoritomus* Bates	Carabidae	island	0.069	0.21
*Pterostichus haptoderoides* (Tschitscherin)	Carabidae	island	0.034	0.49
*Chlaenius variicornis* Morawitz		island	0.034	0.49
*Lithochlaenius noguchii* (Bates)		island	0.034	0.49
*Pterostichus samurai* (Lutshnik)	Carabidae	island	0.034	0.48
*Hemicarabus tuberculosus* (Dejean et Boisduval)		island	0.034	0.48
*Oiceoptoma thoracicum* (Linnaeus)		island	0.034	0.47
*Pterostichus prolongatus* Morawitz	Carabidae	island	0.031	0.74
***Synuchus* spp. Gyllenhal**	**Carabidae**	**lakeshore**	**0.827**	**0.001**
***Pterostichus thunbergi* Morawitz**	**Carabidae**	**lakeshore**	**0.806**	**0.001**
*Geotrupes laevistriatus* Motschulsky	Geotrupidae	lakeshore	0.477	0.56
***Leptocarabus arboreus* (Lewis)**	**Carabidae**	**lakeshore**	**0.355**	**0.001**
*Trichotichnus longitarsis* Morawitz	Carabidae	lakeshore	0.075	0.58
*Pterostichus orientalis* (Motschulsky)	Carabidae	lakeshore	0.065	0.50
*Damaster blaptoides* Kollar	Carabidae	lakeshore	0.065	0.50
*Cychrus morawitzi Gehin*		lakeshore	0.065	0.49
*Leptocarabus opaculus* (Putzeys)	Carabidae	lakeshore	0.032	1.00

The abundance and species richness of carrion beetles at the island site were higher than those at the lakeshore site (*p* < 0.001; Figs 2B and 3B; Tables 1 and 2). The abundance of the two body size groups (medium and large species) were higher at the island site than at the lakeshore site (*p* < 0.001; Fig. 2B; Table 1). The species richness of the two body size groups were also higher at the island site than at the lakeshore site (medium: *p* = 0.04; large: *p* = 0.003). The mean species diversity index was higher at the island site than at the lakeshore site (*p* = 0.005; Fig. 4B; Table 3).

For dung beetles, the abundance (*p* < 0.001) and species richness (*p* = 0.008) at the island site were higher than those at the lakeshore site (Figs 2C, 3C; Tables 1 and 2). Although the abundance and species richness of small species were similarly higher at the island site than at the lakeshore site (*p* < 0.001; Figs 2C, 3C; Tables 1and 2), those were not the case for large species (abundance: *p* = 0.78, species: *p* = 0.90; Fig. 2C, 3C; Tables 1 and 2). The mean species diversity index was higher at the island site than at the lakeshore site (*p* < 0.001; Fig. 4C; Table 3).

**Figure 5.**
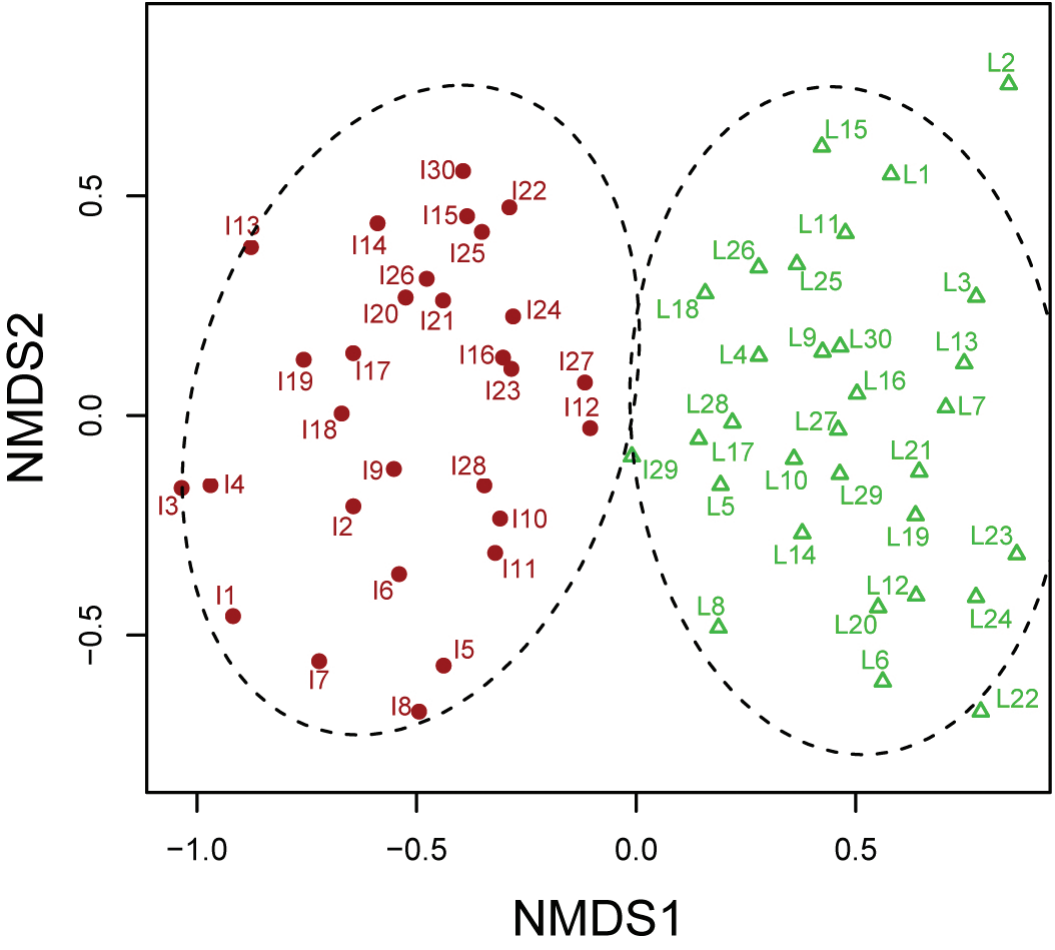
Non-metric multidimensional scaling ordination of each plot of the island and lakeshore sites. From I1 to I30: plots at the island site; from L1 to L30: plots at the lakeshore site. The differences of color and symbol represent the result of clustering.

NMDS and cluster analysis identified a significant difference in species composition between the island and lakeshore sites (Fig. 5). In addition, according to indicator species analysis, indicator species were different between the island and lakeshore sites. Indicator species analysis showed that *Caccobius jessoensis* Harold; *Onthophagus ater* Waterhouse; *Liatongus phanaeoides* (Westwood); *Copris ochus* Motschulsky; *Eusilpha japonica* (Motschulsky); *Silpha perforata* Gebler and *Pterostichus leptis* Bates had significant IndVals for the island site (Table 4). Conversely, Synuchus spp. Gyllenhal; *Pterostichus thunbergi* Morawitz and *Leptocarabus arboreus* (Lewis) had significant IndVals for the lakeshore site.

## Discussion

The current study demonstrated that an increase in sika deer at Lake Toya significantly changed both the abundance and species richness of beetle species belonging to three different taxonomic groups. The abundance and diversity index of carabid beetles at the island site were significantly lower than that at the lakeshore site (Figs 2, 4). In addition, we observed lower species richness and diversity indices at the island site; however they were not statistically significant. As carabid beetles utilize the forest floor as a habitat, the decline of understory vegetation because of overbrowsing is likely to result in the alteration of micro-climate conditions of their habitat, such as a decrease in humidity and an increase in light availability, and a higher mortality by predation (Rooney and Waller 2003, Melis et al. 2007, Cerda et al. 2015). For example, Melis et al. (2007) reported that shade tolerant and hygrophilous carabid species were negatively affected by moose browsing as browsing resulted in a decrease of bilberry coverage and changed the humidity and light intensity. Although we did not measure vegetation and other environmental variables, a significant decline in understory cover has been reported at the island site (Takahashi and Kaji 2001; Fig. 1B).

Conversely, carrion and dung beetles responded positively to deer overabundance, with abundance, species richness and the diversity index higher at the island site (Figs 2–4). Adult and larvae dung beetles utilize the faces of mammals as their main food resource (Andresen and Laurance 2007, Nichols et al. 2009). Hence, deer overabundance at the island site is likely to produce a greater quantity of deer faces, thereby facilitating a more abundant food source for dung beetles as compared to the lakeshore site. Indeed, Kanda et al. (2005) reported a positive relationship between dung beetle abundance and deer density. In addition, as adult and larvae carrion beetles utilize animal carcasses as a food resource (Scott 1998, Dekeirsschieter et al. 2011), deer overabundance may provide greater abundance of food resources for carrion beetles. In support of these assertions, it has similarly been reported that a deer carcass increases the activity of necrophagous beetles (Melis et al. 2004).

Although small carabid species were not affected by deer overabundance, medium and large carabid species were negatively affected by deer overabundance (note: large carabid species were not sampled at the island site). This result suggests that larger carabid species are more sensitive to changing habitat condition than smaller species (Magura et al. 2006, Jelaska and Durbes˘ić 2009). Because the understory vegetation protects these species against extreme microclimate conditions (Ikeda et al. 2005), understory decline because of large herbivore overbrowsing results in a harsh ground floor environment for larger carabid species (Melis et al. 2007). Indeed, a higher susceptibility of larger species to other environmental changes, including habitat fragmentation, deforestation and urbanization, has been reported (Magura et al. 2006, Fujita et al. 2008).

Although the abundance of small dung beetle species was significantly higher at the island site than at the lakeshore site, that of large species did not differ between the two sites. This result suggests that small species are likely to favor a deer abundant environment. In contrast to our results, in Japan, Koike et al. (2014) observed that small species did not prefer environments with higher deer density. One possible reason for the difference would be the difference of dung usage among study species. Koike et al. (2014) found that small species tended to be dwellers which simply lay their eggs into the dung on the ground (Camberfort and Hanski 1991). Therefore, drying because of sunlight exposure, which results from deer overbrowsing, negatively affects these species. Conversely, in our study, the majority of small species were tunnelers, which are species burying dung under the ground before oviposition (Camberfort and Hanski 1991). Because of dung burying, tunnelers are not influenced by the drying of the ground surface.

In our study, NMDS showed a difference in species composition between the island and lakeshore sites (Fig. 5). In addition, indicator species analysis suggested that whereas beetle assemblage at the lakeshore site was characterized by carabid beetle species, that at the island site was characterized by carrion and dung beetle species (Table 4). These results, along with other reported findings indicate a biological homogenization of insect communities at the island site (McKinney and Lockwood 1999). In our study, large herbivore overabundance is likely to cause the homogenization of the beetle assemblage through the increased number of carrion and dung beetles and the decreased number of carabid beetles (Figs 2–4). Moreover, our findings suggest a shift in body size in insect communities; as the number of deer increase, insect communities are more likely to be dominated by smaller individuals.

Although our study brings a valuable contribution, the survey data is limited in its ability for generalizing the current results. Indeed, since the data used in this study was obtained from short term surveys with a limited number of samples (see also the species accumulation curves in Suppl. material 5: Figure 1), our results would provide only partial support for our hypotheses. Thus, further longitudinal studies must be needed to understand the ecological impacts of deer overabundance on insect communities more comprehensively.

## Conclusions

In the present study, evidence is provided that an increase in deer population altered species richness, abundance and diversity of beetles within three different taxonomical groups. Whether such changes affect the ecosystem functions provided by these beetles is unknown. Nevertheless, the observed change in compositions of the three taxonomic beetles groups raises the potential that ecosystem functions may be altered through cascading effects (Larsen et al. 2005, Rouabah et al. 2014). To conserve and maintain overall forest ecosystems, an investigation into the responses of other taxonomic species, including birds or amphibians, to the rapid increase in deer population and its impacts on ecosystem functions is required.

## Supplementary materials

Supplementary material 1 Table 1 Data type: Data table Explanation note: A list of beetle species observed in our study. This dataset is made available under the Open Database License (http://opendatacommons.org/licenses/odbl/1.0/). The Open Database License (ODbL) is a license agreement intended to allow users to freely share, modify, and use this Dataset while maintaining this same freedom for others, provided that the original source and author(s) are credited.Taichi Iida, Masashi Soga, Shinsuke Koike

Supplementary material 2 Table 2 Data type: Data table Explanation note: Estimated and observed values of beetle abundance at the island and lakeshore sites. This dataset is made available under the Open Database License (http://opendatacommons.org/licenses/odbl/1.0/). The Open Database License (ODbL) is a license agreement intended to allow users to freely share, modify, and use this Dataset while maintaining this same freedom for others, provided that the original source and author(s) are credited.Taichi Iida, Masashi Soga, Shinsuke Koike

Supplementary material 3 Table 3 Data type: Data table Explanation note: Estimated and observed values of beetle species richness at the island and lakeshore sites. This dataset is made available under the Open Database License (http://opendatacommons.org/licenses/odbl/1.0/). The Open Database License (ODbL) is a license agreement intended to allow users to freely share, modify, and use this Dataset while maintaining this same freedom for others, provided that the original source and author(s) are credited.Taichi Iida, Masashi Soga, Shinsuke Koike

Supplementary material 4 Table 4 Data type: Data table Explanation note: Estimated and observed values of beetle diversity (Shannon-Wiener diversity index) at the island and lakeshore sites. This dataset is made available under the Open Database License (http://opendatacommons.org/licenses/odbl/1.0/). The Open Database License (ODbL) is a license agreement intended to allow users to freely share, modify, and use this Dataset while maintaining this same freedom for others, provided that the original source and author(s) are credited.Taichi Iida, Masashi Soga, Shinsuke Koike

Supplementary material 5 Figure 1 Data type: Figure Explanation note: Species accumulation curves for carabid, carrion and dung beetles at the island (blue lines) and lakeshore sites (red lines). This dataset is made available under the Open Database License (http://opendatacommons.org/licenses/odbl/1.0/). The Open Database License (ODbL) is a license agreement intended to allow users to freely share, modify, and use this Dataset while maintaining this same freedom for others, provided that the original source and author(s) are credited.Taichi Iida, Masashi Soga, Shinsuke Koike
