# Supplementary material for: Effects of an increase in population of sika deer on beetle communities in deciduous forests
Source: Zookeys. 2016 Oct 19;(625):67–85. doi: 10.3897/zookeys.625.9116 (PMC5096363; doi:10.3897/zookeys.625.9116)
Supplement: Supplementary material 1 — Table 1 [file zookeys-625-067-s001.doc]

**Supplementary Table 1**

|  | Taxonomic group | Body size ^a^ | Island | Lakeshore |
| --- | --- | --- | --- | --- |
| *Pterostichus haptoderoides* (Tschitscherin) | Carabid beetle | Small | 2 | 0 |
| *Synuchus spp.* Gyllenhal | Carabid beetle | Small | 4 | 145 |
| *Trichotichnus longitarsis* Morawitz | Carabid beetle | Small | 4 | 6 |
| *Chlaenius pallipes* Gebler | Carabid beetle | Medium | 28 | 6 |
| *Chlaenius variicornis* Morawitz | Carabid beetle | Medium | 1 | 0 |
| *Cychrus morawitzi* Géhin | Carabid beetle | Medium | 0 | 3 |
| *Hemicarabus tuberculosus*(Dejean et Boisduval) | Carabid beetle | Medium | 1 | 0 |
| *Leptocarabus opaculus* (Putzeys) | Carabid beetle | Medium | 0 | 1 |
| *Lithochlaenius noguchii* (Bates) | Carabid beetle | Medium | 1 | 0 |
| *Pterostichus leptis* Bates | Carabid beetle | Medium | 80 | 0 |
| *Pterostichus orientalis* (Motschulsky) | Carabid beetle | Medium | 0 | 2 |
| *Pterostichus planicollis* (Motschulsky) | Carabid beetle | Medium | 4 | 0 |
| *Pterostichus prolongatus* Morawitz | Carabid beetle | Medium | 9 | 1 |
| *Pterostichus samurai* (Lutshnik) | Carabid beetle | Medium | 1 | 0 |
| *Pterostichus thunbergii* Morawitz | Carabid beetle | Medium | 58 | 362 |
| *Pletostichus yoritomus* Bates | Carabid beetle | Medium | 2 | 0 |
| *Damaster blaptoides* Kollar | Carabid beetle | Large | 0 | 2 |
| *Leptocarabus arboreus* (Lewis) | Carabid beetle | Large | 0 | 13 |
| *Nicrophorus quadripunctatus* Kraatz | Carrion beetle | Medium | 10 | 6 |
| *Oiceoptoma thoracium* (Linné) | Carrion beetle | Medium | 1 | 0 |
| *Silpha perforata* Gebler | Carrion beetle | Medium | 49 | 10 |
| *Eusilpha japonica* (Motschulsky) | Carrion beetle | Large | 69 | 2 |
| *Caccobius jessoensis* Harold | Dung beetle | Small | 1196 | 6 |
| *Liatongus phanaeoides* (Westwood) | Dung beetle | Small | 18 | 0 |
| *Onthophagus ater* Waterhouse | Dung beetle | Small | 1218 | 273 |
| *Copris ochus* Motschulsky | Dung beetle | Large | 9 | 0 |
| *Geotrupes laevistriatus* Motschulsky | Dung beetle | Large | 157 | 161 |
| ^a^ Carabid and Carrion beetles: Small (<10mm), Medium (>10mm, <20mm) or Large (>20mm); Dung beetles: Small (<10mm) or Large (>10mm) | | | | |
