# Supplementary material for: Effects of an increase in population of sika deer on beetle communities in deciduous forests
Source: Zookeys. 2016 Oct 19;(625):67–85. doi: 10.3897/zookeys.625.9116 (PMC5096363; doi:10.3897/zookeys.625.9116)
Supplement: Supplementary material 2 — Table 2 [file zookeys-625-067-s002.doc]

**Supplementary Table 2**

|  | Abundance (mean ± SE) | | | | | | | | | | |
| --- | --- | --- | --- | --- | --- | --- | --- | --- | --- | --- | --- |
|  | Lakeshore | | | | |  | Island | | | | |
|  | Estimated value | |  | Observed value | |  | Estimated value | |  | Observed value | |
|  | mean | SE |  | mean | SE |  | mean | SE |  | mean | SE |
| Carabid beetles | 18.03 | 1.04 |  | 18.03 | 2.40 |  | 9.93 | 1.07 |  | 9.93 | 2.54 |
| small group | 1.19 | 1.62 |  | 0.20 | 0.11 |  | 2.63 | 1.98 |  | 0.20 | 0.15 |
| medium group | 12.50 | 1.05 |  | 12.50 | 1.72 |  | 6.17 | 1.09 |  | 6.17 | 1.55 |
| large group | NA | NA |  | 0.50 | 0.12 |  | NA | NA |  | 0.00 | 0.00 |
|  |  |  |  |  |  |  |  |  |  |  |  |
| Carrion beetles | 0.60 | 1.27 |  | 0.60 | 0.17 |  | 4.37 | 1.29 |  | 4.37 | 0.89 |
| medium group | 0.11 | 2.09 |  | 0.53 | 0.16 |  | 3.70 | 2.11 |  | 2.00 | 0.69 |
| large group | 1.00 | 1.00 |  | 0.07 | 0.05 |  | 1.00 | 1.00 |  | 2.30 | 0.55 |
|  |  |  |  |  |  |  |  |  |  |  |  |
| Dung beetles | 14.67 | 1.05 |  | 14.67 | 2.02 |  | 86.60 | 1.05 |  | 86.60 | 10.23 |
| small group | 9.30 | 1.06 |  | 9.30 | 1.72 |  | 81.07 | 1.07 |  | 81.07 | 9.84 |
| large group | 5.37 | 1.08 |  | 5.37 | 0.76 |  | 5.53 | 1.12 |  | 5.53 | 1.29 |
