# Supplementary material for: Effects of an increase in population of sika deer on beetle communities in deciduous forests
Source: Zookeys. 2016 Oct 19;(625):67–85. doi: 10.3897/zookeys.625.9116 (PMC5096363; doi:10.3897/zookeys.625.9116)
Supplement: Supplementary material 3 — Table 3 [file zookeys-625-067-s003.doc]

**Supplementary Table 3**

|  | Species richness (mean ± SE) | | | | | | | | | | |
| --- | --- | --- | --- | --- | --- | --- | --- | --- | --- | --- | --- |
|  | Lakeshore | | | | |  | Island | | | | |
|  | Estimated value | |  | Observed value | |  | Estimated value | |  | Observed value | |
|  | mean | SE |  | mean | SE |  | mean | SE |  | mean | SE |
| Carabid beetles | 1.80 | 1.15 |  | 1.80 | 0.17 |  | 1.57 | 1.22 |  | 1.57 | 0.21 |
| small group | 0.13 | 1.65 |  | 0.13 | 0.06 |  | 0.07 | 2.38 |  | 0.07 | 0.05 |
| medium group | 1.23 | 1.18 |  | 1.23 | 0.11 |  | 1.50 | 1.25 |  | 1.50 | 0.21 |
| large group | NA | NA |  | 0.43 | 0.10 |  | NA | NA |  | 0.00 | 0.00 |
|  |  |  |  |  |  |  |  |  |  |  |  |
| Carrion beetles | 0.43 | 1.32 |  | 0.43 | 0.11 |  | 1.37 | 1.37 |  | 1.37 | 0.18 |
| medium group | 0.07 | 2.03 |  | 0.37 | 0.10 |  | 0.60 | 2.11 |  | 0.77 | 0.15 |
| large group | 1.00 | 1.00 |  | 0.07 | 0.05 |  | 1.00 | 1.00 |  | 0.60 | 0.09 |
|  |  |  |  |  |  |  |  |  |  |  |  |
| Dung beetles | 2.13 | 1.13 |  | 2.13 | 0.09 |  | 3.27 | 1.17 |  | 3.27 | 0.14 |
| small group | 1.17 | 1.18 |  | 1.17 | 0.08 |  | 2.33 | 1.23 |  | 2.33 | 0.09 |
| large group | 0.97 | 1.20 |  | 0.97 | 0.03 |  | 0.93 | 1.30 |  | 0.93 | 0.12 |
