# Supplementary material for: Effects of an increase in population of sika deer on beetle communities in deciduous forests
Source: Zookeys. 2016 Oct 19;(625):67–85. doi: 10.3897/zookeys.625.9116 (PMC5096363; doi:10.3897/zookeys.625.9116)
Supplement: Supplementary material 4 — Table 4 [file zookeys-625-067-s004.doc]

**Supplementary Table 4**

|  | Shannon diversity (mean ± SE) | | | | | | | | | | |
| --- | --- | --- | --- | --- | --- | --- | --- | --- | --- | --- | --- |
|  | Lakeshore | | | | |  | Island | | | | |
|  | Estimated value | |  | Observed value | |  | Estimated value | |  | Observed value | |
|  | mean | SE |  | mean | SE |  | mean | SE |  | mean | SE |
| Carabid beetles | 0.84 | 0.11 |  | 0.84 | 0.10 |  | 0.54 | 0.15 |  | 0.54 | 0.11 |
|  |  |  |  |  |  |  |  |  |  |  |  |
| Carrion beetles | 0.07 | 0.07 |  | 0.07 | 0.05 |  | 0.35 | 0.10 |  | 0.35 | 0.09 |
|  |  |  |  |  |  |  |  |  |  |  |  |
| Dung beetles | 0.83 | 0.05 |  | 0.83 | 0.06 |  | 1.17 | 0.07 |  | 1.17 | 0.04 |
