# Supplementary figures and images for: Effects of an increase in population of sika deer on beetle communities in deciduous forests
Source: Zookeys. 2016 Oct 19;(625):67–85. doi: 10.3897/zookeys.625.9116 (PMC5096363; doi:10.3897/zookeys.625.9116)

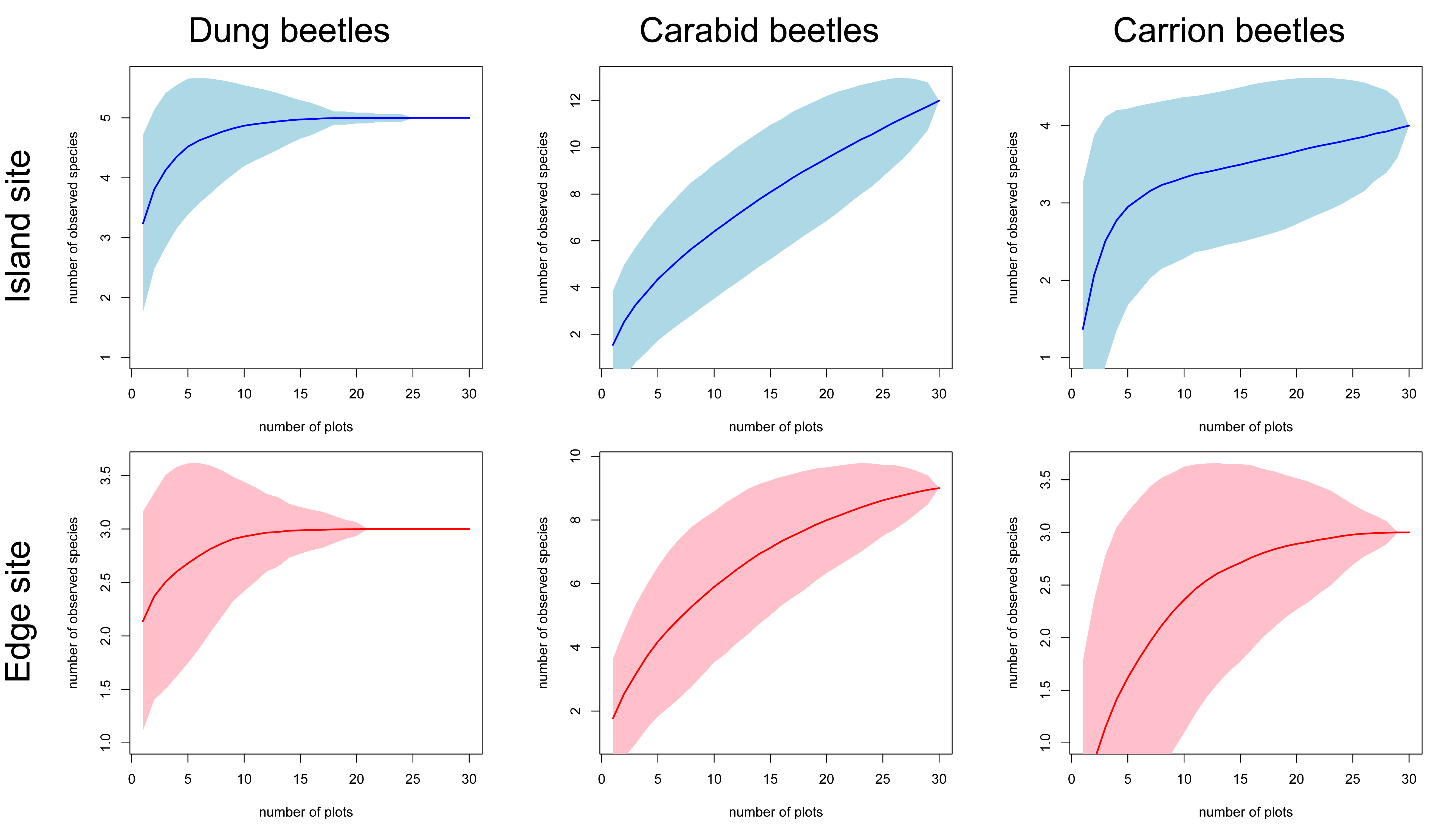


**Supplementary Figure 1**

Supplement: Supplementary material 5 — Figure 1 [file zookeys-625-067-s005.doc]
